# Relationship Between Emotional Intelligence and Health Behaviours among University Students: The Predictive and Moderating Role of Gender

**DOI:** 10.1155/2018/7058105

**Published:** 2018-06-04

**Authors:** Romualdas Malinauskas, Audrone Dumciene, Saule Sipaviciene, Vilija Malinauskiene

**Affiliations:** ^1^Lithuanian Sports University, Department of Health, Physical and Social Education, Kaunas, Lithuania; ^2^Lithuanian Sports University, Department of Applied Biology and Rehabilitation, Sporto 6, 44221 Kaunas, Lithuania

## Abstract

This study investigated the role of gender as a potential predictor of health behaviour and potential moderator of the relationship between emotional intelligence and health behaviour. This cross-sectional study included 1214 students (597 males and 617 females). Data were collected using the Schutte Self-Report Inventory and the Health Behaviour Checklist. Stepwise multiple regression analysis was executed with the components of health behaviour as the dependent variables to examine the predictive value of the emotional intelligence indicators as the independent variables. Gender predicted all categories of health behaviours. Only one indicator of emotional intelligence, appraisal, predicted the Accident Control and Traffic Risk Taking categories. The emotional intelligence indicator of social skills emerged only as a predictor of Wellness Maintenance and Enhancement in university students. Gender moderates the relationship between all emotional intelligence indicators and health behaviour components except the relationship between Appraisal and Substance Risk Taking and the relationship between Utilization and traffic risk taking.

## 1. Introduction

Recently, researchers have focused on the emotional intelligence links to improve psychological and physical health [1–6]. Previous meta-analyses emphasised that the link between trait emotional intelligence and mental health is important [7, 8]. Martins et al. [7] and Schutte et al. [8] found that emotional intelligence was significantly and positively related to physical, psychosomatic, and mental health.

Generally speaking, emotional intelligence can be defined as the ability to perceive, control, and evaluate emotions [6, 9]. Emotional intelligence can be differentiated in line with two different methods of assessment: ‘trait' emotional intelligence (TEI) and ‘ability' emotional intelligence (AEI). First, TEI is considered as a cluster of emotion-related self-perceptions/dispositions evaluated via self-report [3, 10]. Second, AEI is a direct assessment of actual proficiency in perceiving, understanding, using, and managing emotion through measures of maximal performance [11]. In the present study the TEI approach was used because the measurement of trait EI is much more straightforward [10] and there already exist several widely used instruments for its assessment, e.g., [12, 13]. The theoretical underpinnings of trait EI, along with an operational definition of the construct and its precise location in established trait hierarchies, are presented in Petrides and Furnham [14] study. TEI refers to a constellation of behavioural dispositions and self-perceptions concerning one's ability to recognise, process, and utilise emotionally laden information. The authors [15] consider that TEI has great potential, at least in terms of application, in social contexts. Among the TEI measures, for instance, TEIQue is based on a sound theoretical foundation that predicts neurophysiological outcomes and has demonstrated strong psychometric properties in numerous samples [15]. The TEIQue has a better coverage of the TEI sampling domain and is the only scale where psychometric shortcomings are absent [15, 16]. However, TEIQue is not tested and not validated in Lithuanian-speaking sample. Another tool, i.e., Schutte Self-Report Inventory (SSRI, based on the original model of Salovey and Mayer [17]), has been used for the present study because it is tested and validated in Lithuanian-speaking sample [18].

Previous research has shown that emotional intelligence acts as a facilitator of positive health practices [9]. In particular, emotionally intelligent individuals are more likely to maintain proactive self-care practices (e.g., regular exercise, healthy diet [6, 9]). Some research has suggested that emotional intelligence may also be related to enhanced coping resources and more adaptive habitual coping behaviours that carry added health benefits [17]. Examples of health-related behaviour include a nutritious diet, controlled efforts to maintain regular exercise, and adequate sleep, all of which have been associated with increased positive mood and better health-related quality of life [6].

Studies on TEI and health have suggested that associations between TEI and behaviours may promote physical health [6]. A study by Saklofske et al. [19] showed small but significant correlations between TEI and measures of healthy diet and exercise. Furthermore, Saklofske et al. [20] found that TEI is related to self-reports of exercise behaviour but not to exercise attitudes. Several scholars have analysed the link between emotional intelligence and psychological health. They suggest a direct link between emotional intelligence and psychological health [21–23].

Health behaviour, rather than physical or mental health, has been chosen as the dependent variable because the leading causes of morbidity and death today are related to chronic stress, unhealthy lifestyle, and health-related behaviours [6]. The health risks are associated with smoking, alcohol abuse, and traffic accidents [24]. Gochman [25] in the Handbook of Health Behaviour Research defines them as ‘behaviour patterns, actions and habits that relate to health maintenance, to health restoration and to health improvement' (p. 3). Behaviours within this definition include self-directed health behaviours (e.g., smoking, alcohol consumption, and substance risk taking behaviour).

The present research makes a novel contribution to the scientific domain of emotional intelligence because, based on a large university students sample, the study extends previous results to provide more indicators of health behaviour* (wellness maintenance, traffic risk taking, accident control,* and* substance risk taking) *testing gender as a potential predictor and potential moderator of the relationship between emotional intelligence and health behaviour. There is a lack of emotional intelligence research with university student population showing associations between perceived (trait) emotional intelligence and health behaviours because it was only possible to find two studies [26, 27] showing associations between perceived (trait) emotional intelligence and health behaviours among college students. Specifically, Pettit et al. [27] included only measures of health behaviours, conceptualised as coping responses to stress while Goldman et al. [26] did not use measures of health-risk behaviours but included only measures of students' visits to a health centre. The present study includes various categories of health behaviours (wellness maintenance, traffic risk taking, accident control, and substance risk taking). Some studies have reported that females had higher emotional intelligence scores than males [28]. Some research has indicated that university students' health behaviour may vary with gender.* For instance, men engaged in more risky health behaviours than women [29, 30].* When health behaviour varies with gender it promptly points to a potential moderator variable.

The present study had two major goals. The first was to delimit specific emotional intelligence indicators (optimism, appraisal, social skills, and utilisation) that best predict categories of health behaviours (wellness maintenance, traffic risk taking, accident control, and substance risk taking). The first directional hypothesis was that the university students' specific emotional intelligence indicators and health-risk behaviours differed in respect to gender; i.e., women had higher levels of emotional intelligence indicators than men and women engaged in the risk behaviours less than men. This hypothesis was based on earlier study which reported that females had higher emotional intelligence indicators scores than males [28] and on meta-analysis of 150 studies in which the risk taking tendencies of male and female participants were compared [31]. The results of meta-analysis clearly supported the idea that male participants are more likely to take risks and health-risk behaviours than female participants.

A second aim was to test the role of gender as a potential predictor of health behaviour and potential moderator of the relationship between emotional intelligence and health behaviour. The second directional hypothesis was that at high levels of specific emotional intelligence indicators, women reported higher healthy behaviour (wellness maintenance, less traffic risk taking, accident control, and less substance risk taking) than men at the same level of emotional intelligence indicators. This hypothesis was based on a study [23] which reported that higher levels of emotional intelligence indicators among women in comparison with the levels of emotional intelligence indicators among men contribute more significantly to women's health behaviour than to men's health behaviour.

## 2. Method

### 2.1. Study Design and Participants

A cross-sectional study was conducted. Multistage sampling method was used. A sample of seven universities from Lithuania was randomly selected (every second) out of 15 in state universities in Lithuania. Three groups were randomly selected from each faculty (humanities and social and technical sciences). The sample consisted of 1214 first-to-fourth year university students between the age of 19 and 25 (597 males and 617 females) who were enrolled in humanities (34.2%) and social (36.2%) and technical sciences (29.6%) courses. Balanced sample of students of the different degree courses was realized: 26,4% of them were first year, 25,6% of them were second year, 24.7% of them were third year, and 23,3% of them were senior year university students. No statistically significant differences among the students belonging to the different degree courses and different faculties courses were found. The mean age of the participants was 22.36 (SD = 1.86) years. The students participated voluntarily, with no financial incentive.

The researchers presented the study and provided the participants information about the study objectives. Participants completed the questionnaire (described below) during scheduled class time, with no time limit. The researchers received ethical approval to conduct this study. The study was approved by the Committee for Biomedical Sciences Research Ethics of Lithuanian Sports University. This research meets the ethical guidelines, including adherence to the legal requirements of the country where this study was conducted. According to Lithuanian legislation informed consent from the study population (participants over the age of 18) is not required for research, which involves anonymous surveys. Participants were instructed to mark the response “I agree to participate” or “I disagree to participate” (on the survey's first page) to give their consent to participate in the study before beginning the survey.

### 2.2. Measures

#### 2.2.1. Schutte Self-Report Inventory (SSRI)

Emotional intelligence was measured using the SSRI which was validated by Schutte et al. [13]. The SSRI, otherwise known as the EIS (Emotional Intelligence Scale), the SEI (Self-Report Emotional Intelligence), and the Schutte Emotional Intelligence Scale (SEIS), assesses El based on self-report responses. It has been chosen because of the 33 studies included in meta-analytical review [32]; the most common assessments were the Schutte EI Scale (Schutte et al. [13]; n = 12). This scale measures the participants' perception about their emotional skills, both at an intrapersonal and an interpersonal level. It consists of 33 Likert items answered on a five-point scale, where 1 =* strongly disagree*, 2 =* disagree*, 3 =* neutral*, 4 =* agree*, and 5 =* strongly agree*. Psychometric properties have been examined by various authors who agree on the usefulness of this scale as a brief measure of emotional intelligence [14]. In addition, Schutte et al. [13] proved the reliability and validity of this scale, not only for measuring emotional intelligence but also for its effects on people. This instrument is extremely beneficial in the way that it divides emotional intelligence into four separate components [33], namely, using own positive emotional experience (optimism), expression of emotion (appraisal), understanding and analysis of emotion (social skills), and utilisation of emotion (utilisation). We decide to analyse data with subscales and not with global index of emotional intelligence because we would like to get the results of the greatest possible number of emotional intelligence indicators in order to delimit those that best predict health behaviours. The internal consistency for this research was good (*α* = 0.76). The Lithuanian version of the SSRI showed an internal consistency value of 0.79 and a test-retest reliability coefficient of 0.84 for the overall questionnaire [18].

#### 2.2.2. Health Behaviour Checklist (HBC)

The HBC [29] was used to measure health practices consistent with good health. This scale asks about preventative behaviours that are aimed at maintaining or improving health, undertaken by persons who consider themselves to be in good health. Vickers and colleagues [34] reported the multidimensionality of health behaviours, which led to the development of the particular factors reported by this measure. The HBC consists of 40 items, of which 28 are used to assess four health behaviours [35].

On the basis of findings that replicated across several independent samples, Vickers et al. [34] depicted four distinct, replicable dimensions of health behaviour which could be derived from 28 of the 40 items. These are the following four scales of Health Behaviour Checklist (HBC). (1) The Wellness Maintenance and Enhancement dimension consists of items such as ‘I exercise to stay healthy'. (2) The accident control dimension includes items like ‘I fix broken things around my home straight away'. (3) The traffic risk taking dimension consists of items such as ‘I drive after drinking'. This item was reversed scored, as a higher score on the scale actually reflects less traffic risk taking (i.e., healthier driving behaviour). (4) The substance risk taking dimension included items like ‘I do not drink'. Items were scored in a manner such that a lower score on this scale indicates less substance risk tasking (i.e., healthier pattern of substance use). Participants were asked to indicate how well each item described their typical behaviour using a five-point Likert scale ranging from 1 = strongly disagree to 5 = strongly agree. The procedures used to develop the HBC are depicted in Vickers et al. [29], as well as the reliability and validity of the scale. There is also evidence of criterion-referenced validity in comparison with relevant measures [34–36]. The Lithuanian version of the HBC showed an internal consistency value of .67 for the overall questionnaire [37].

Table 1 presents the results of internal consistency and the descriptive statistics of the four factors of those scales from this study.

### 2.3. Statistical Analysis

Descriptive statistics of data were presented. Skewness and kurtosis coefficients were computed for univariate normality analyses purposes, and all values were within ±1. To test gender differences Student's t-test was performed because the data were normally distributed. Correlations were presented as Pearson product moment correlations (two-tailed) for all continuous variables. The requirement for the use of the linear regression of the multicollinearity of the predictors is verified. As no correlation exceeded .70, the assumption of multicollinearity was not violated. To examine the predictive value of gender and the emotional intelligence indicators as the independent variables, stepwise multiple regression analyses were executed with the components of health behaviours.

Hierarchical moderated regression analyses were used to test how gender moderates the relationship between emotional intelligence indicators and health behaviour components. The predictor variables (main effects) were entered in the regression equation in step 1, followed by the 2-way interactions in step 2. The independent variables were centred by standardizing them before the product term was created [38]. The standardized solution was then examined. All of these statistical analyses were conducted using SPSS (Version 19.0).

## 3. Results

Cronbach's alphas, means, and SDs were calculated for each* subscale.*Table 1 introduced the results for the total sample of participants, by gender. All the internal consistency values were within acceptable levels. The results of the independent samples t-tests conducted to determine gender differences are also presented in Table 1. Statistically significant differences (*p* < 0.001) were found between men and women in respect to all the EI indicators and all categories of health behaviours (the categories of the HBC scale).

Statistical frequencies (%) of risk behaviours (smoke, alcohol, and taking chemical substances) between men and women are reported in Table 2.

Our findings indicate that male university students have a lower proportion regarding smoking (41.25%) than males in the general population (73.0%) of that age group in Lithuania [39]. Female university students have also a lower proportion with respect to smoking (13%) than females in the general population (58.4%) of that age group in Lithuania. The percentage of male university students with respect to consumption of alcohol (55.6%) is lower than that of men in the general population (59.8%) of that age group in Lithuania. The percentage of female university students stands at 43.1% compared with the percentage of women (42.8%) in general population of that age group in Lithuania [39].

Pearson correlation coefficients were calculated to analyse the correlation among the gender, emotional intelligence indicators and health behaviours components. The results are summarised in Table 3.

As Table 3 reveals, almost all the emotional intelligence indicators had a significant and positive correlation with the health behaviour components, except for the optimism, appraisal, and utilisation dimensions, which had significant but negative correlations with substance risk taking. Specifically, appraisal did not correlate with Wellness Maintenance and Enhancement; utilisation did not correlate with accident control; social skills did not correlate with substance risk taking. Gender had a significant and positive correlation with all the emotional intelligence indicators and categories of health behaviours. The only strong association force is between gender and traffic risk taking, while the others are statistically significant but do not have a strong correlation.

Prior to the stepwise multiple regression analysis, the relationships between independent variables (SSRI) and the dependent variables (HBC) were explored. Independent variables were significantly related to the health behaviour components which were assumed as candidate predictors and were entered into the stepwise multiple regression analysis.

In all the analyses, gender (1 = male; 2 = female) was included as an independent variable to find whether it predicted health behaviours (the dependent variables were each of the categories of the HBC scale). The results are summarised in Table 4.

Regarding Wellness Maintenance and Enhancement, the prediction model consisted of three predictors and was obtained in three steps,* F*(3,1210) = 9.643, *p* < 0.01, accounting for 5.3% of the variance (*R*^2^ = 0.053). Significant predictors of this model were optimism (*R*^2^= 0.031), social skills (*R*^2^ = 0.014), and gender (*R*^2^ = 0.008).

The emotional intelligence indicators predicted almost equally the two components of health behaviours: Accident Control and Traffic Risk Taking. Regarding accident control the prediction model consisted of two predictors and was obtained in two steps, *F*(2,1211) = 41.637, *p* < 0.001, accounting for 10.3% of the variance (*R*^2^ = 0.103). Significant predictors were gender (*R*^2^ = 0.72), with women assuming more accident control behaviours (mean scores of male and female participants in Table 1) and appraisal (*R*^2^ = 0.031). Regarding traffic risk taking, the prediction model consisted of predictors and was reached in two steps, *F*(2,1211) = 38.746, *p* < 0.001, accounting for 17.7% of the variance (*R*^2^ = 0.177). Significant predictors of this model were also gender (*R*^2^ = 0.151), with men assuming more risk behaviours related to traffic safety (mean scores of male and female participants in Table 1) and appraisal (*R*^2^ = 0.026). Regarding the component substance risk taking, the prediction model consisted of four predictors, *F*(4,1209) = 15.804, *p* < 0.001, accounting for 4.2% of the variance (*R*^2^ = 0.042). Significant predictors of this model were gender (*R*^2^ = 0.006), with men assuming more risk behaviours regarding consumption of substances such as alcohol and tobacco (mean scores of male and female participants are presented in Table 1), appraisal (*R*^2^ = 0.018), optimism (*R*^2^ = 0.005), and utilisation (*R*^2^ = 0.013). The emotional intelligence indicator of social skills emerged as a predictor only for Wellness Maintenance and Enhancement category. Optimism and appraisal best predicted these types of behaviours.

We used hierarchical moderated regression analyses to test the study hypothesis about moderating role of gender in the relationship between emotional intelligence indicators and health behaviour categories among university students. A significant change in *R*^2^ in the second step of regression analyses and a significant b weight for the interaction between the moderator (gender) and emotional intelligence indicators indicate the presence of moderator effects. In general, Gender was found to be moderator of the relationship between all emotional intelligence indicators and health behaviour components with two exceptions (Table 5).

As shown in Table 5, the interaction between gender and utilisation was not significant. Change in *R*^2^ in the second step of regression analyses was not significant for substance risk taking behaviour when in the first step of regression analysis we entered appraisal and gender, followed by the interaction between these variables in the second step. Graphs of the moderating effect of gender on the relationship between all emotional intelligence indicators and health behaviour components are presented in Supplementary Material 1. For men, as emotional intelligence indicators increased there was a much sharper increase in Substance Risk Taking than for women (see Supplementary Material 1). Also, at high levels of emotional intelligence indicators, women reported higher Wellness Maintenance and Enhancement and accident control than men at the same level of emotional intelligence indicators, and the converse occurred at low levels of emotional intelligence indicators.

## 4. Discussion

This study sought to test the role of gender as a potential predictor of health behaviour and as a potential moderator of the relationship between emotional intelligence and health behaviour among university students. One of our objectives was to get the results with the greatest possible number of emotional intelligence indicators in order to single out those that best predict health behaviours. In the present study emotional intelligence was measured using the SSRI, which is beneficial in the way that not only global index of emotional intelligence could be computed but also four separate components could be assessed [33]. In addition, we also examined relations between emotional intelligence and health behaviours in the present study, utilising the HBC, a scale especially tailored to assess healthy behaviours.

Regarding the relation between emotional intelligence and health behaviours, only one or two unhealthy behaviours, such as alcohol and tobacco consumption, were tested in past studies [23, 24, 40] and negative relation between total emotional intelligence and smoking and drinking was found. In the present study, additional to the results for the risk behaviours of substance consumption, results concerning traffic risk taking and accident control were also obtained. Regarding health behaviours, the results of the regression analyses showed that three emotional intelligence components predicted substance risk taking: appraisal, optimism, and utilisation. The only emotional intelligence component that predicted Accident Control and Traffic Risk Taking was appraisal. Only two emotional intelligence components, optimism and appraisal, were related to Wellness Maintenance and Enhancement.

Moreover, gender was as a predictor of health behaviours, with men getting higher values than the women in the health-risk behaviours: men seem to display more risk behaviours regarding consumption of substances (e.g., alcohol and tobacco) as well as traffic safety. The findings of the present could be explained by Tsaousis and Nikolaou [23] argument that increased levels of emotional intelligence have an important role on health functioning and health behaviours.

The first hypothesis that women had higher levels of emotional intelligence indicators than men and women engaged in the risk behaviours less than men was confirmed. The results of this study are in line with another study [28] which found students gender differences for emotional intelligence indicators. It was also confirmed that female university students engaged in the risk behaviours less than male university students. The results of the present research could be explained by growing body of literature which suggests that men's and women's health is differentially affected by psychosocial, structural, and behavioural determinants [30, 41].

Statistical frequencies of risk behaviours between male and female university students between the age of 19 and 25 were compared. These frequencies also were compared with those of the general population of that age group in Lithuania. Percentage of prevalence of alcohol in the present university students sample for males and females was similar to percentage of prevalence of alcohol in the general population of that age group. Percentage of prevalence of smoke in the present university students sample for males and females was different from percentage of prevalence of smoke in the general population of that age group and therefore results of the present sample cannot be generalized to population of the youth of that age.

Regarding the associations between the emotional intelligence indicators and the health behaviour component Wellness Maintenance and Enhancement, the results showed that almost all the emotional intelligence indicators, except appraisal, were positively related to this health behaviour component. A possible explanation for this finding could be that high emotional intelligence should lead to more successful and efficient self-regulation through health-related behaviours, thereby supporting help-seeking and the maintenance of health regimens (e.g., avoiding fattening foods, avoiding sedentary lifestyle [6, 9]).

Regarding accident control, all the emotional intelligence components, except for utilisation, had a positive relation with this health behaviour component. Results of the present study were similar to those received by authors Fernández-Abascal and Martín-Díaz [21], although in our study, not all relations between emotional intelligence indicators and health behaviour components were significant.

Regarding traffic risk taking, all the emotional intelligence indicators had a positive relation with this health behaviour component. The findings of our work do not coincide with the results of other researchers, particularly Fernández-Abascal and Martín-Díaz [24], who found negative relations. One possible reason for this discrepancy could be the different measures used for emotional intelligence indicators. The second reason is that traffic risk taking could be influenced by risk behaviour in the family that may have been learned in the family and by socioeconomic status of the university students (belonging to less or more affluent classes could influence risk behaviour). However, the variables of the socioeconomic status of the students and data on risk behaviour in the family are missing in the present research.

Regarding substance risk taking, all the emotional intelligence components, except for social skills, had a significant and negative relation with this health behaviour component indicating that higher scores on this emotional intelligence category correlated with less use of these substances. This finding was consistent with similar emotional intelligence research [23, 24, 42] establishing lower emotional intelligence as a key predictor of substance use. They argued that, among youth, high emotional intelligence was related to lower probabilities of involvement with alcohol and cigarette smoking.

A second aim was to test the role of gender as a potential predictor of health behaviour and potential moderator of the relationship between emotional intelligence and health behaviour. Our prediction that gender moderates the relationship between emotional intelligence indicators and health behaviour categories was confirmed as the interactions between gender and optimism were significant for all four categories of health behaviours (Wellness Maintenance and Enhancement, accident control, traffic risk taking, and substance risk taking), the interactions between gender and Social skills were significant for all four all categories of health behaviours, the interactions between gender and appraisal were significant for three categories of health behaviours, and the interactions between gender and appraisal were significant for three categories of health behaviours. Gender was not only strong predictor of health-risk behaviours but also strong moderator, with men getting higher values than the women in substance risk taking behaviours at high levels of emotional intelligence indicators.

The second hypothesis that, at high levels of specific emotional intelligence indicators, women reported higher healthy behaviour (wellness maintenance, less traffic risk taking, accident control, and less substance risk taking) than men at the same level of emotional intelligence indicators were partially confirmed because only two exceptions were found: the first exceptions for the relationship between Appraisal and substance risk taking and the second for the relationship between Utilization and traffic risk taking. As expected, at high levels of emotional intelligence indicators, female university students reported higher health behaviour (Wellness Maintenance and Enhancement and accident control) than male university students at the same level of emotional intelligence indicators, and the converse occurred at low levels of emotional intelligence indicators. This provides support for the notion that high levels of emotional intelligence indicators contribute to women's health behaviour. It is in line with arguments that high emotional intelligence is related to lower probabilities of involvement with health-risk behaviours (for instance, with alcohol and cigarette smoking) [23, 42]. It could be in line with arguments by Dawson et al. [30] that health behavioural choices that individuals make early in their lives influence their health behaviours later on, with this relationship being influenced by gender but there are no data on risk behaviour in the family that may have been learned in the family.

To summarise, in general gender predicted all categories of health behaviours in university students. Only one indicator of emotional intelligence, appraisal, predicted the Accident Control and Traffic Risk Taking categories. The emotional intelligence indicator of social skills was only a predictor of Wellness Maintenance and Enhancement in university students. Finally, we indicate that gender appears as a predictor of health behaviours, with men receiving higher values than the women in the health-risk behaviours. All the emotional intelligence indicators, except for social skills, are involved in substance risk taking behaviour. Gender moderates the relationship between all emotional intelligence indicators and health behaviour components except the relationship between Appraisal and Substance Risk Taking and the relationship between Utilization and traffic risk taking. However, our analysed population represented a selected population of university students at age of 19–25 and not population of the youth of Lithuania of that age, so no reliable conclusions about the general population of that age group can be drawn.

*Limitations and Strengths. *The present study has several limitations. First, data analyses were based on correlations, and the study design makes it difficult to draw cause-and-effect conclusions. Second, the research was carried out with self-report measures; hence it is plausible that social desirability may have affected the responses, particularly if it is taken into account that the respondents completed the questionnaire in an academic context.* The third limitation of the present research is that SSRI has been used instead of the *Trait Emotional Intelligence Questionnaire (TEIQue), which is the only scale where psychometric shortcomings are absent [16, 32]. Fourth, the variables of the socioeconomic status of the university students are missing and belonging to less or more affluent classes could influence certain risk behaviours. Fifth, there are no data on risk behaviour in the family (alcohol and smoking) and therefore this data cannot be generalized with respect to the components of emotional intelligence or gender with respect to these behaviours that may have been learned in the family. The sixth limitation is that selected sample of the participants of the present study represented a selected population of university students and not of the youth of that age.

Among the strengths is the fact that the current study extends previous results with large university students' population and provides more indicators of health behaviour (wellness maintenance, traffic risk taking, accident control, and substance risk taking) showing gender not only as a potential predictor of health behaviour but also as potential moderator of the relationship between emotional intelligence and health behaviour. This present study brings together two areas of research, namely, emotional intelligence and health behaviour. Both areas of research individually have received considerable attention but together have been rarely analysed.

## 5. Conclusions

The findings of this study have both theoretical and practical implications. From a theoretical perspective, gender was identified as predictor of all categories of health behaviours (including substance risk taking); thus our findings contribute to a better understanding of health behaviours in this context. It was also found that all the emotional intelligence indicators, except for social skills, are involved in substance risk taking behaviour. Identifying predictors of substance risk taking is of great significance for development of these characteristics among students; thus, our findings also have practical implications. Educational institutions can create opportunities for the development of emotional intelligence inside and outside the classes. Our findings suggest that persons with lower emotional intelligence using substances such as alcohol and tobacco more can be due to the education provided at home and at school. Persons may not have been supported adequately at home or in educational institutions regarding characteristics of emotional intelligence, namely, using own positive emotional experience (optimism), expression of emotion (appraisal), understanding and analysis of emotion (social skills), and utilisation of emotion (utilisation). The development of these characteristics must be more supported both at home and in educational institutions. We think that the results of the present study can be applied in educational institutions for stretching of development of emotional intelligence.

## Figures and Tables

**Table 1:** Cronbach's alphas, means, and SDs of the variables.

Scale (number of items)	Cronbach Alpha	Total sample	SD	Men	SD	Women	SD	*t*
		*N* = 1214		*N = *597		*N *= 617		
		*M*		*M*		*M*		
Schutte Self-Report Inventory (SSRI)								
Optimism (14)	0.80	3.33	0.55	2.91	0.47	3.69	0.52	−27.44^*∗∗∗*^
Social Skills (9)	0.70	3.16	0.53	2.76	0.51	3.56	0.55	−26.29^*∗∗∗*^
Appraisal (6)	0.87	3.27	0.58	2.94	0.51	3.58	0.45	−23.48^*∗∗∗*^
Utilization (5)	0.76	2.92	0.68	2.62	0.62	3.21	0.61	−16.84^*∗∗∗*^
Health behavior checklist (HBC)								
Wellness (11)	0.69	3.23	0.43	3.15	0.39	3.29	0.49	−5.52^*∗∗∗*^
Accident Control (6)	0.71	3.24	0.63	3.07	0.57	3.41	0.63	−9.74^*∗∗∗*^
Traffic Risk Taking (7)	0.66	2.88	0.35	3.05	0.37	2.72	0.25	18.37^*∗∗∗*^
Substance Risk Taking (4)	0.84	2.60	0.72	2.64	0.71	2.53	0.72	2.68^*∗∗*^

Notes. *p* calculated in Student's t test. ^*∗∗∗*^*p* < 0.001 (two-tailed); ^*∗∗*^*p* < 0.01 (two-tailed); ^*∗*^*p* < 0.05 (two-tailed).

Wellness is Wellness Maintenance and Enhancement.

**Table 2:** Statistical frequencies (%) of risk behaviors (smoke, alcohol, and taking chemical substances) between men and women.

	Total (*N* = 1214)		Men (*N* = 597)		Women (*N *= 617)	
	n	%	n	%	n	%
Smoking						
no	641	52.8	157	26.3	484	78.4^*∗∗∗*^
yes	326	26.9	246	41.2	80	13.0
undecided	247	20.3	194	32.5	53	08.6

Alcohol drinking						
no	291	24.0	108	18.1	183	29.7^*∗∗*^
yes	598	49.3	332	55.6	266	43.1
undecided	325	26.8	157	26.3	168	27.2

Taking chemical substances						
no	280	23.1	129	21.6	151	24.5
yes	742	61.1	368	61.6	374	60.6
undecided	192	15.8	100	16.8	92	14.9

Notes. *p* calculated in Chi-square test. ^*∗∗∗*^*p* < 0.001 (two-sided); ^*∗∗*^*p* < 0.01 (two-sided); ^*∗*^*p* < 0.05 (two-sided) between men and women.

Taking chemical substances – taking chemical substances which might injure health (e.g., food additives, drugs, and stimulants).

Yes: agree and strongly agree, No: disagree and strongly disagree, and Undecided: neither agree or disagree.

**Table 3:** Pearson correlations between the gender, emotional intelligence indicators, and health behavior components.

	Wellness	Accident Control	Traffic Risk Taking	Substance Risk Taking	Gender
Optimism	.177^*∗∗*^ (*p* = .000)	.101^*∗∗*^ (*p* = .000)	.187^*∗∗*^ (*p* = .000)	−.061^*∗*^ (*p* = .041)	.624^*∗∗*^ (*p* = .000)
Social Skills	.146^*∗∗*^ (*p* = .000)	.089^*∗∗*^ (*p* = .002)	.211^*∗∗*^ (*p* = .000)	.008 (*p* = .774)	.604^*∗∗*^ (*p* = .000)
Appraisal	.047 (*p* = .100)	.105^*∗∗*^ (*p* = .000)	.083^*∗∗*^ (*p* = .004)	−.070^*∗*^ (*p* = .015)	.559^*∗∗*^ (*p* = .000)
Utilization	.120^*∗∗*^ (*p* = .000)	.024 (*p* = .404)	.155^*∗∗*^ (*p* = .000)	.−.058^*∗*^ (*p* = .042)	.435^*∗∗*^ (*p* = .000)
Gender	.159^*∗∗*^ (*p* = .000)	.269^*∗∗*^ (*p* = .000)	.388^*∗∗*^ (*p* = .000)	.077^*∗∗*^ (*p* = .008)	1

Total sample *N* = 1214, ^*∗∗∗*^*p* < 0.001 (two-tailed); ^*∗∗*^*p* < 0.01 (two-tailed); ^*∗*^*p* < 0.05 (two-tailed).

Wellness is Wellness Maintenance and Enhancement.

**Table 4:** Stepwise multiple regression analysis results.

Model	*R*	*R* ^2^	*R* ^2^	*R* ^2^	*F *(df)	*β*	*β*	*t*
			adjusted	change			standardized	
Dependent variables: components of the Health behavior checklist (HBC)								

Independent variables: components of the SSRI questionnaire								

Dependent variable: Wellness								

Model 1:	.177	.031	.030	.031	39.030 (1212)^*∗∗∗*^			
Optimism						125	.177	6.248^*∗∗∗*^

Model 2:	.213	.045	.044	.014	17.702 (1211)^*∗∗∗*^			
Optimism						.213	.302	7.379^*∗∗∗*^
Social Skills						.133	.172	4.207^*∗∗∗*^

Model 3:	.230	.053	.050	.011	9.643 (1210)^*∗∗*^			
Optimism						.176	.249	5.655^*∗∗∗*^
Social Skills						.152	.197	4.571^*∗∗∗*^
Gender						.101	.113	3.105^*∗∗∗*^

Dependent variable: Accident Control								

Model 1:	.269	.072	.072	.072	94.667 (1212)^*∗∗∗*^			
Gender						.337	.269	9.730^*∗∗∗*^

Model 2:	.321	.103	.102	.031	41.637 (1211)^*∗∗∗*^			
Gender						.486	.388	11.809^*∗∗∗*^
Appraisal						−.230	−.212	−6.453^*∗∗∗*^

Dependent variable: Traffic Risk Taking								

Model 1:	.388	.151	.150	.151	215.208 (1212)^*∗∗∗*^			
Gender						385	.388	14.670^*∗∗∗*^

Model 2:	.421	.177	.176	.026	38.746 (1211)^*∗∗∗*^			
Gender						.494	.498	15.831^*∗∗∗*^
Appraisal						−.168	−.196	−6.225^*∗∗∗*^

Dependent variable: Substance Risk Taking								

Model 1:	.077	.006	.005	.006	7.173 (1212)^*∗∗*^			
Gender						.110	.077	2.678^*∗∗*^

Model 2:	.156	.024	.023	.018	22.885 (1211)^*∗∗*^			
Gender						.241	.168	4.915^*∗∗∗*^
Appraisal						−.203	−.164	−4.784^*∗∗∗*^

Model 3:	.171	.029	.027	.005	6.336 (1210)^*∗*^			
Gender						.190	.133	3.589^*∗∗∗*^
Appraisal						−.279	−.225	−5.363^*∗∗∗*^
Optimism						−.128	−.112	− 2.517^*∗*^

Model 4:	.205	.042	.039	.013	15.804 (1209)^*∗∗∗*^			
Gender						.205	.143	3.882^*∗∗∗*^
Appraisal						−.287	−.232	−5.536^*∗∗∗*^
Optimism						−.230	−.203	−4.066^*∗∗∗*^
Utilization						−.152	−.145	−3.975^*∗∗∗*^

Total sample *N* = 1214; ^*∗∗∗*^*p* < 0.001; ^*∗∗*^*p* < 0.01; ^*∗*^*p* < 0.05. In all the analyses, gender was entered as an independent variable to determine whether it predicted health behaviors.

**Table 5:** Moderated regression analyses.

Independent variable	Wellness			Accident Control			Traffic Risk Taking			Substance Risk Taking		
	*B*	*SE*	*ΔR* ^*2*^	*B*	*SE*	*ΔR* ^*2*^	*B*	*SE*	*ΔR* ^*2*^	*B*	*SE*	*ΔR* ^*2*^
Step 1: Main effects			.02^*∗∗*^			.08^*∗∗*^			.22^*∗∗*^			.01^*∗*^
Gender	.09^*∗∗*^	.03		.40^*∗∗*^	.04		−.32^*∗∗*^	.02		−.13^*∗∗*^	.05	
Optimism	.05	.27		−.11^*∗∗*^	.04		−.01	.02		.04	.05	
Step 2: Two-way interaction			.10^*∗∗*^			.08^*∗∗*^			.01^*∗∗*^			.05^*∗∗*^
Gender	.08^*∗∗*^	.03		.40^*∗∗*^	.04		−.32^*∗∗*^	.02		−.13^*∗∗*^	.05	
Optimism	.16^*∗∗*^	.03		.04	.04		.02	.02		−.10^*∗*^	.05	
Gender x Optimism	.59^*∗∗*^	.05		.75^*∗∗*^	.07		.15^*∗∗*^	.04		−.71^*∗∗*^	.09	
Intercept	2.46^*∗∗*^	.08		2.42^*∗∗*^	.11		3.29^*∗∗*^	.06		3.21^*∗∗*^	.14	

Step 1: Main effects			.02^*∗∗*^			.10^*∗∗*^			.22^*∗∗*^			.02^*∗∗*^
Gender	.14^*∗∗*^	.03		.43^*∗∗*^	.10		−.34^*∗∗*^	.02		−.19^*∗∗*^	.05	
Social Skills	−.05	.03		−.21^*∗∗*^	.04		.03	.02		.16^*∗∗*^	.04	
Step 2: Two-way interaction			.06^*∗∗*^			.05^*∗∗*^			.01^*∗∗*^			.05^*∗∗*^
Gender	.15^*∗∗*^	.03		.45^*∗∗*^	.04		−.34^*∗∗*^	.02		−.20^*∗∗*^	.04	
Social Skills	.01	.03		−.14^*∗∗*^	.04		.05^*∗∗*^	.02		.08	.04	
Gender x Social Skills	.43^*∗∗*^	.05		.57^*∗∗*^	.07		.16^*∗∗*^	.04		−.65^*∗∗*^	.08	
Intercept	2.88^*∗∗*^	.08		2.92^*∗∗*^	.11		3.21^*∗∗*^	.08		2.73^*∗∗*^	.13	

Step 1: Main effects			.02^*∗∗*^			.10^*∗∗*^			.22^*∗∗*^			.02^*∗∗*^
Gender	.15^*∗∗*^	.03		.49^*∗∗*^	.04		−.34^*∗∗*^	.02		−.24^*∗∗*^	.05	
Appraisal	−.05^*∗*^	.03		−.23^*∗∗*^	.04		.01	.02		.20^*∗∗*^	.04	
Step 2: Two-way interaction			.01^*∗∗*^			.01^*∗∗*^			.02^*∗∗*^			.00
Gender	.15^*∗∗*^	.03		.48^*∗∗*^	.04		−.34^*∗∗*^	.02		−.24^*∗∗*^	.05	
Appraisal	−.02	.03		−.17^*∗∗*^	.04		.05^*∗∗*^	.02		.18^*∗∗*^	.05	
Gender x Appraisal	.18^*∗∗*^	.05		.27^*∗∗*^	.07		.19^*∗∗*^	.04		−.11^*∗∗*^	.09	
Intercept	2.99^*∗∗*^	.07		3.03^*∗∗*^	.12		3.19^*∗∗*^	.06		2.39^*∗∗*^	.14	

Step 1: Main effects			.02^*∗∗*^			.08^*∗∗*^			.22^*∗∗*^			.02^*∗∗*^
Gender	.10^*∗∗*^	.03		.40^*∗∗*^	.04		−.34^*∗∗*^	.02		−.18^*∗∗*^	.05	
Utilization	.03	.02		−.11^*∗∗*^	.03		.01	.02		.12^*∗∗*^	.03	
Step 2: Two-way interaction			.13^*∗∗*^			.14^*∗∗*^			.00			.16^*∗∗*^
Gender	.10^*∗∗*^	.03		.40^*∗∗*^	.04		−.34^*∗∗*^	.02		−.18^*∗∗*^	.04	
Utilization	.11	.02		.02	.03		.01	.02		−.04	.03	
Gender x Utilization	.50^*∗∗*^	.04		.75^*∗∗*^	.05		.01	.02		−.92^*∗∗*^	.06	
Intercept	2.63^*∗∗*^	.06		2.45^*∗∗*^	.08		3.35^*∗∗*^	.05		3.12^*∗∗*^	.10	

*Note. B* represents the unstandardized regression coefficients for each step in the regression equation. *N =* 1214. ^*∗*^*p* < .05; ^*∗∗*^*p* < .01.

## Data Availability

The data used to support the findings of this study are available from the corresponding author upon request.

## Supplementary Materials

Supplementary Materials Supplementary Material 1: graphs of the moderating effect of gender on the relationship between all emotional intelligence indicators and health behaviour components.
