# Supplementary material for: Relationship Between Emotional Intelligence and Health Behaviours among University Students: The Predictive and Moderating Role of Gender
Source: Biomed Res Int. 2018 Jun 4;2018:7058105. doi: 10.1155/2018/7058105 (PMC6008781; doi:10.1155/2018/7058105)
Supplement: Supplementary Materials — Supplementary Material 1: graphs of the moderating effect of gender on the relationship between all emotional intelligence indicators and health behaviour components. [file 7058105.f1.docx]

Supplementary Material 1: Graphs of the moderating effect of gender on the relationship between all emotional intelligence indicators and health behaviour components

Figure 1.1. Graph of the moderating effect of gender on the relationship between Optimism and Wellness

*Note.* Wellness is Wellness Maintenance and Enhancement.

Figure 1.2. Graph of the moderating effect of gender on the relationship between Optimism and Accident Control

Figure 1.3. Graph of the moderating effect of gender on the relationship between Optimism and Traffic Risk Taking

Figure 1.4. Graph of the moderating effect of gender on the relationship between Optimism and Substance Risk Taking

Figure 2.1. Graph of the moderating effect of gender on the relationship between Social Skills and Wellness

*Note.* Wellness is Wellness Maintenance and Enhancement.

Figure 2.2. Graph of the moderating effect of gender on the relationship between Social Skills and Accident Control

Figure 2.3. Graph of the moderating effect of gender on the relationship between Social Skills and Traffic Risk Taking

Figure 2.4. Graph of the moderating effect of gender on the relationship between Social Skills and Substance Risk Taking

Figure 3.1. Graph of the moderating effect of gender on the relationship between Appraisal and Wellness

*Note.* Wellness is Wellness Maintenance and Enhancement.

Figure 3.2. Graph of the moderating effect of gender on the relationship between Appraisal and Accident Control

Figure 3.3. Graph of the moderating effect of gender on the relationship between Appraisal and Traffic Risk Taking

Figure 3.4. No moderating effect of gender on associations between Appraisal and Substance Risk Taking

Figure 4.1. Graph of the moderating effect of gender on the relationship between Utilization and Wellness

*Note.* Wellness is Wellness Maintenance and Enhancement.

Figure 4.2. Graph of the moderating effect of gender on the relationship between Utilization and Accident Control

Figure 4.3. No moderating effect of gender on associations between Utilization and Traffic Risk Taking

Figure 4.4. Graph of the moderating effect of gender on the relationship between Utilization and Substance Risk Taking
